# Eustachian tube dysfunction: A diagnostic accuracy study and proposed diagnostic pathway

**DOI:** 10.1371/journal.pone.0206946

**Published:** 2018-11-08

**Authors:** Matthew E. Smith, Yemisi Takwoingi, Jon Deeks, Cuneyt Alper, Manohar L. Bance, Mahmood F. Bhutta, Neil Donnelly, Dennis Poe, James R. Tysome

**Affiliations:** 1 Cambridge Ear Institute, University of Cambridge, Cambridge, United Kingdom; 2 Institute of Applied Health Research, University of Birmingham, Edgbaston, Birmingham, United Kingdom; 3 Children’s Hospital of Pittsburgh, University of Pittsburgh Medical Center, Pittsburgh, Pennsylvania, United States of America; 4 Brighton & Sussex University Hospitals NHS Trust, Brighton, United Kingdom; 5 Boston Children’s Hospital, Boston, Massachusetts, United States of America; University of Connecticut Health Center, UNITED STATES

## Abstract

**Background and aims:**

Eustachian tube dysfunction (ETD) is a commonly diagnosed disorder of Eustachian tube opening and closure, which may be associated with severe symptoms and middle ear disease. Currently the diagnosis of obstructive and patulous forms of ETD is primarily based on non-specific symptoms or examination findings, rather than measurement of the underlying function of the Eustachian tube. This has proved problematic when selecting patients for treatment, and when designing trial inclusion criteria and outcomes. This study aims to determine the correlation and diagnostic value of various tests of ET opening and patient reported outcome measures (PROMs), in order to generate a recommended diagnostic pathway for ETD.

**Methods:**

Index tests included two PROMs and 14 tests of ET opening (nine for obstructive, five for patulous ETD). In the absence of an accepted reference standard two methods were adopted to establish index test accuracy: expert panel diagnosis and latent class analysis. Index test results were assessed with Pearson correlation and principle component analysis, and test accuracy was determined. Logistic regression models assessed the predictive value of grouped test results.

**Results:**

The expert panel diagnosis and PROMs results correlated with each other, but not with ET function measured by tests of ET opening. All index tests were found to be feasible in clinic, and acceptable to patients. PROMs had very poor specificity, and no diagnostic value. Combining the results of tests of ET function appeared beneficial. The latent class model suggested tympanometry, sonotubometry and tubomanometry have the best diagnostic performance for obstructive ETD, and these are included in a proposed diagnostic pathway.

**Conclusions:**

ETD should be diagnosed on the basis of clinical assessment and tests of ET opening, as PROMs have no diagnostic value. Currently diagnostic uncertainty exists for some patients who appear to have intermittent ETD clinically, but have negative index test results.

## Introduction

The Eustachian tube (ET) is a dynamic tubular structure which normally only opens to facilitate gaseous pressure regulation of the middle ear, but otherwise remains closed to prevent the transmission of sound, pressure, nasopharyngeal secretions and pathogens from the nose to the ear. Eustachian tube dysfunction (ETD) is a frequently applied diagnosis, used when abnormal ET function is believed to underlie any of a wide range of symptoms or middle ear (ME) abnormalities. Two distinct forms of the disorder are recognised, representing different ends of the spectrum of ET function: 1) obstructive ETD (OETD), in which tubal opening or patency is reduced, and 2) patulous ETD (PETD), in which the ET is too open[1]. Even within these two subtypes, the condition remains heterogeneous in terms of aetiology and presentation, and it has been suggested that some patients with OETD have either predominantly active dysfunction (failure of muscle-controlled opening), or passive dysfunction (failure of pressure-related opening)[2].

Most ETD is diagnosed on the basis of the reported clinical history, examination of the ear and nasopharynx, and routine tests such as tympanometry. More objective tests of ET function have been proposed, with renewed efforts in validating and developing tests in recent years due to the need for outcome measures for emerging medical and surgical interventions. There is no reference (gold standard) method to diagnose OETD or PETD, and the clinical diagnosis of an experienced clinician has been the default reference standard for many years. In order to introduce objective outcome measures to trials of ETD interventions, tympanometric classification, Valsalva or breathing-related ME pressure change, patient reported outcome measures (PROMs) and many other tests have been proposed as potential diagnostic markers of ETD.

Clinicians and researchers wishing to advance the diagnosis of ETD beyond clinical opinion currently have difficulty determining which tests should be used. Efforts have been made to describe the accuracy of tests that measure ET opening, and to develop PROMs[3], but diagnostic accuracy research is complex in conditions such as ETD where the disorder is poorly defined and lacks a reference method for diagnosis.

Test accuracy is measured by assessing the performance of the evaluated (index) test against the reference standard, within the same population of subjects with the suspected target disorder[4]. To conduct test accuracy research in conditions such as ETD, a new reference standard can be synthesised. This may be done through: 1) a predefined rule (composite reference standard); 2) consensus among experts (panel diagnosis); 3) a statistical model based on the collected test results (latent class analysis)[5, 6].

A composite reference standard for ETD is not possible due to the lack of evidence relating existing tests to the condition.

An expert panel can be used as a reference standard by establishing the presence or absence of a disorder in individual patients. The expert panel method is suited to conditions such as ETD where multiple sources of information such as patient characteristics, symptoms, examination findings and other test results must be interpreted in a judicious way to reach a diagnosis, particularly in the absence of an unequivocal definition of the disorder[5].

Latent class analysis uses a statistical model to combine different test results from each patient to construct a reference standard. The technique aims to determine the latent variable (diagnosis) on the basis of manifest variables (the test results). Latent class models perform well if there are large numbers of index tests, as they increase the possible test combinations and available degrees of freedom. However, these models require test results to be independent, and so not all possible ETD tests can be modelled together.

Both diagnosis and test accuracy are heavily influenced by the situation and cohort in which they are assessed. Most tests for ETD have not been investigated in a representative clinical setting where patients have an uncertain diagnosis. In contrast, most tests have been assessed in a case-control design study, with cases selected to represent a single, often more severe form of ETD are matched with healthy controls. These two factors tend to overestimate test accuracy, as in practice the task is to differentiate subjects who are disease positive from subjects who have many of the same symptoms, but do not have the disorder[7, 8]. In addition, most tests have been assessed for diagnostic accuracy in isolation, missing the potential improvement that may be gained by combining test results, either informally as part of the diagnostic process, or mathematically in a risk calculator.

Finally, the status of clinical assessment as the current default reference standard diagnosis for ETD has not been challenged. Instead, tests that do not support the clinical reference diagnosis have been deemed inaccurate. The converse is of course possible. Without the assessment of multiple and varied tests in a single cohort, it has not been possible to establish the correlation between different diagnostic methods, and the accuracy of clinical diagnosis.

This prospective diagnostic accuracy study aims to explore the relationship between results from a wide range of different methods for diagnosing ETD, and to establish their accuracy compared to a reference standard, generated using both latent class analysis and panel diagnosis. We discuss the clinical implementation of these tests, and a recommended diagnostic pathway.

## Methods and materials

Ethical approval was obtained from the UK Research Ethics Service. Written consent was obtained from all participants.

### Participants

A single cohort of non-consecutive patients with symptoms or examination findings suggestive of ETD were selectively recruited from ENT outpatient clinics at Addenbrooke’s Hospital, Cambridge, between November 2016 and May 2017, according to the following criteria:

#### Inclusion criteria

≥18 years old

One of the following findings (formalising current clinical practice):

- at least 2 symptoms of ETD*
- AND/OR tympanic membrane retraction
- AND/OR negative pressure tympanogram (≤-100daPa)

*list in S1 Table, and based upon a literature review.

#### Exclusion criteria

- Otoscopic findings that may prevent use of the full range of index tests (otitis media with effusion, tympanic membrane perforation^$^, cholesteatoma, discharging or infected ear)
- Cardiac pacemaker (incompatible with the sonotubometry speaker)
- Patients with cleft palate or craniofacial abnormality (due to concerns regarding their ability to perform the full range of tests, or to obtain interpretable results)
- Inability to consent or poor understanding of written English

### Assessments

Recruited patients initially underwent an assessment equivalent to the current standard of care in clinics without a specialist interest in ETD. Patients then underwent assessment with various tests for ETD (index tests).

#### Standard-of-care assessment (4 components)

1. A focused, standardised clinical history of the patient’s presenting complaint, screening for symptoms and medical history associated with ETD.
2. Otoscopic examination: recording retraction, atelectasis, and other relevant features of the tympanic membrane.
3. Pure tone audiometry with air and bone conduction thresholds.
4. Middle ear pressure and compliance measured via tympanometry.

#### Index tests

Two forms of ET function test were assessed as index tests: objective and semi-objective measures of ET opening, and symptom-based PROMS. Tests of ET opening for OETD (9 tests) and PETD (5 tests) were performed according to optimal methods previously identified[9, 10] or standard procedure (PETD tests). Details of the tests of ET opening, their various methods and the repeats performed can be found in Table 1. Two PROMs were completed by patients: the CETDA[11] and ETDQ-7[12]. Composite measures of ET function were also assessed: the 5- and 7-item Eustachian Tube Scores (ETS and ETS-7) were calculated for each case, combining patient-reported symptoms with tubomanometry results in a simple numerical scale, with lower scores indicative of OETD[13].

**Table 1:** Tests of ET opening assessed, including manoeuvres used and number of repeats.

ET Function Test	Method (no. of measurements)	Diagnostic outcome measured (unit)	Other variables recorded (unit)*	ET function tested
**Obstructive ETD**				
Tympanometry	Patient at rest (1)	ME pressure (daPa) Tympanic admittance (ml)	-	Active opening (ME pressure also influenced by mucosal gas exchange)
Nine-step test	3 dry swallows, +400daPa in EAC (1) 3 dry swallows, - 400daPa in EAC (1)	ME pressure change after ±400daPa (daPa) ME pressure change after equilibration (daPa)	-	Active opening
Patient-reported assessment	Valsalva (3) Dry Toynbee (3)	Patient sensation (yes/no)	-	Passive opening (Valsalva) Passive and active opening (Toynbee)
Observation of the tympanic membrane	Valsalva (3) Dry Toynbee (3)	Tympanic membrane movement (yes/no)	Strength and speed of TM movement (weak/strong, fast/slow)	Passive opening (Valsalva) Passive and active opening (Toynbee)
Tubo-tympano-aerodynamic-graphy (TTAG)	Valsalva (3) Dry Toynbee (3)	Peak positive and negative change in EAC pressure (daPa)	NP pressure (daPa)	Passive opening (Valsalva) Passive and active opening (Toynbee)
Continuous impedance	Valsalva (3) Dry Toynbee (3)	Peak positive and negative change in tympanic impedance (ml)	NP pressure (daPa)	Passive opening (Valsalva) Passive and active opening (Toynbee)
Sonotubometry	Dry swallow (5)	Peak EAC sound pressure level (dB) Duration of EAC recording (ms)	-	Active opening
Tubomanometry	Wet swallow, 300daPa (1) Wet swallow, 400daPa (1) Wet swallow, 500daPa (1)	Peak EAC pressure change (daPa) R value (No unit)	-	Passive and active opening
Tuboimpedance	Wet swallow, 300daPa (1) Wet swallow, 400daPa (1) Wet swallow, 500daPa (1)	Peak tympanic impedance change (ml)	-	Passive and active opening
**Patulous ETD**				
Observation of the tympanic membrane	10 seconds heavy breathing (1)	Breathing synchronous tympanic membrane movement (yes/no)	-	Passive closure
TTAG	10 seconds heavy breathing (1)	Breathing synchronous change in EAC pressure (daPa)	-	Passive closure
Continuous impedance	10 seconds heavy breathing (1)	Breathing synchronous change in tympanic impedance (ml)	-	Passive closure
Sonotubometry	10 seconds heavy breathing (1)	Breathing synchronous change in EAC sound pressure level (dB)	-	Passive closure
Tubomanometry	Wet swallow, 300daPa (1) Wet swallow, 400daPa (1) Wet swallow, 500daPa (1)	R value (No unit)	-	Passive and active opening


ME = middle ear, NP = nasopharyngeal, EAC = external auditory canal, TTAG = Tubo-tympano-aerodynamic-graphy. In addition for each test the following were recorded; time taken to complete the test (min/sec), patient reported difficulty to complete the test (Likert scale 1–10, 1 = none) and discomfort during the test (Likert scale1-10, 1 = none).

All participants underwent all OETD and PETD tests in a single session, with a single assessor (MS). Although previous work suggested fatigue and test interactions were minimal[10], the index tests were applied in a partly-randomised manner; tympanometry and the nine step test were always applied first to prevent residual middle ear pressure influencing results, while the order of the other tests was randomised using an online random sequence generator[14]. PROMs were also completed in a randomised order.

To assess feasibility in clinical practice, and the burden on the patient, for each test we also recorded: time taken to complete the test from start of patient instruction to end of data recording (min/sec); patient reported difficulty in completing the test (Likert scale 1–10, 1 = none, 10 = almost impossible); and discomfort during the test (Likert scale 1–10, 1 = none, 10 = almost unbearable).

Data were recorded on paper forms and in Microsoft Excel.

### Expert panel diagnosis

A six-person international expert panel was recruited from clinicians working and publishing in the field of ETD. The panel’s role was to provide a reference standard diagnosis of ETD, through the consensus opinion of experts. The six experts were divided into two groups, forming Panel A (MBa, MBh, ND) and Panel B (CA, DP, JT).

To reduce assessor burden, study participants were randomly allocated into two groups for independent review by one of the two panels. To ensure that each panel had a similar case mix, allocation was stratified according to original clinic diagnosis (OETD / PETD / alternate diagnosis). As part of the allocation process, a subgroup of 20 patients was allocated to both panels to assess inter-panel agreement. The random sampling was done by YT using PROC SURVEYSELECT in the SAS statistical package.

Each patient’s history, examination and audiometry findings were presented to the panel on a separate case summary slide (Microsoft Powerpoint). Summary slides included information available in ENT outpatients (including the results of investigations (e.g. nasendoscopy or imaging), and the response to treatments initiated in the community (e.g. nasal steroid spray, or betahistine). Index test results were not presented to the panel to prevent incorporation bias falsely inflating test accuracy[8], with the exception of tympanometry, as this test is established and widely available in current ENT practice.

Initially, each panel member assessed cases independently and without conferring. They were asked to diagnose ETD if they felt it was present, even if some symptoms, or the primary complaint, were thought to be due to another condition. Panel members were asked to consider three possible diagnoses: OETD, PETD, or an alternate diagnosis.

**Fig 1:**
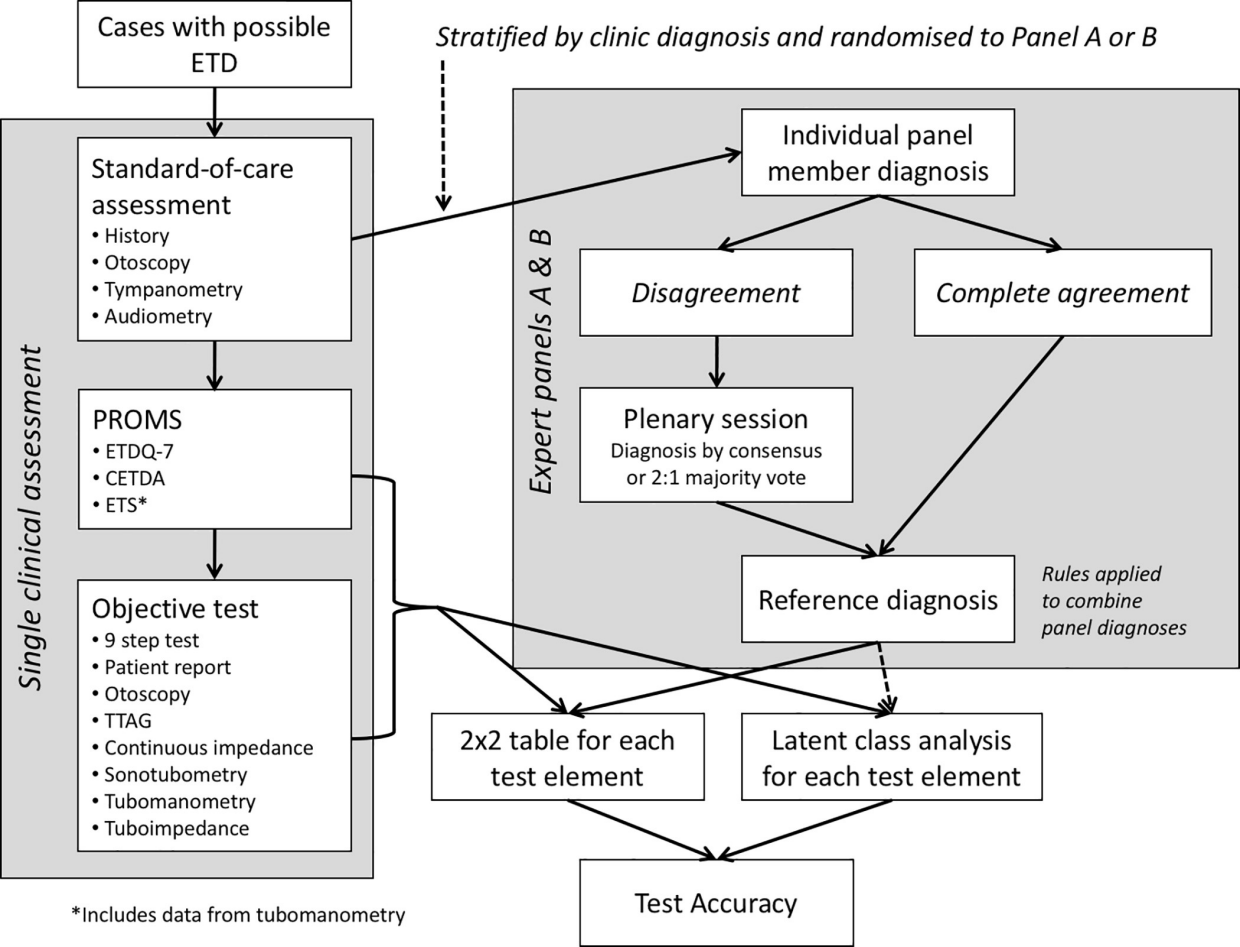
Flow chart demonstrating the data collection process and generation of a reference standard, with subsequent analysis to assess test accuracy.

Results from individual expert assessment were analysed, and where all three experts in a panel agreed on a diagnosis, this diagnosis was assigned. Disagreements were subsequently discussed at a panel teleconference (Fig 1) where panels A&B met separately. Summary data for each study participant were reviewed and panel members discussed the reasons for their individual diagnoses. The panels were directed to reach a consensus diagnosis if possible. Where consensus could not be reached, the diagnosis was made by a majority vote of 2–1.

Rules were set in advance to assign a diagnosis in the event that there was disagreement between the panels A&B when diagnosing the subset of 20 shared cases:

1. Diagnoses made via individual diagnoses in agreement were assigned over those with initial disagreement
2. Diagnoses made at discussion via consensus were assigned over those made by a 2:1 vote
3. If rule 1 or 2 could not be applied, (i.e. there were conflicting diagnoses from panels A&B, made in an equivalent manner) the diagnosis was recorded as indeterminate.

Agreement between panels for the 20 shared cases was measured with Cohen’s Kappa.

### Statistical analysis and modelling

Raw test data were initially analysed using descriptive statistics in accordance with diagnostic criteria described in the literature and previous work[9, 10].

#### Assessment of correlation and latent variables

Principle component analysis (PCA) was used to detect common latent variables within the test outcomes. PCA was performed using continuous or ordinal variables from each of the tests and PROMs, and the panel diagnosis. To confirm adequacy of sampling, the Kaiser-Meyer-Olkin (KMO) value was calculated (>0.6 adequate), and Bartlett’s Test of Sphericity performed. Components with an Eigenvalue <1 were disregarded. Pairwise correlations for the function tests, PROMs and panel diagnosis were performed by creating a correlation matrix (bivariate Pearson correlation). PCA and correlation were performed with SPSS 25 (IBM, Armonk, New York, USA). An explanatory description of PCA and latent class analysis (see below) is included in the Supporting Information (S1 File).

#### Test accuracy based on expert panel as reference standard

Estimation of the accuracy of individual tests: Using the panel diagnosis, 2x2 tables were generated for each index test. For tests measured on a continuous or ordinal scale, sensitivity and specificity were calculated at pre-specified and novel diagnostic thresholds (Table 2, column 3). As test repetitions were employed to improve repeatability of findings[10], novel thresholds using the repetitions were generated to allow interpretation. Receiver operating characteristic (ROC) curves were also plotted and the area under the curve (AUC) estimated. Thresholds at which sensitivity was maximised and the false positive rate was minimised were identified on ROC curves. Test performance was also summarised using Youden's index (*J*, where *J* = sensitivity + specificity– 1) where 1 indicates a perfect test and 0 indicates no diagnostic value[15]. Accuracy was assessed for tests of both patulous and obstructive ETD.

**Table 2:** Summary of test results for ETD, with different interpretation methods.

Test	Parameter	Interpretation for OETD	Median	% cases	Expert Panel Diagnosis			
				positive	AUC (95% CI)	Sensitivity (95% CI)	Specificity (95% CI)	Youden’s index *J*
**Obstructive ETD**						n = 57 OETD	n = 57 non-OETD	
Tympanometry	Patient at rest	Raw data–ME pressure in daPa	-11	-	0.66 (.56-.76)	-	-	-
		<-50 daPa	-	23.1	-	35.1 (22.9–48.8)	91.2 (80.7–97.0)	0.26
		<-100 daPa	-	15.4	-	26.3 (15.5–39.6)	98.2 (90.6–100.0)	0.25
Nine-step test	+400daPa	Raw data—daPa change	-15	-	0.60 (.50-.71)	-	-	-
		<5DaPa change	-	25.0	-	31.6 (19.9–45.2)	80.7 (68.0–89.9)	0.12
		<10DaPa change	-	35.6	-	45.6 (32.3–59.3)	73.7 (60.3–84.4)	0.19
	+ve equilibration	Raw data—daPa change	6	-	0.62 (.52-.72)	-	-	-
		<5DaPa change	-	35.6	-	38.6 (25.9 52.4)	66.7 (52.9–78.5)	0.05
		<10DaPa change	-	51.0	-	63.2 (49.3–75.5)	61.4 (47.5–74.0)	0.25
	- 400daPa	Raw data—daPa change	9	-	0.46 (.36-.55)	-	-	-
		<5DaPa change	-	47.1	-	59.6 (38.9–66.0)	57.9 (44.0–70.8)	0.11
		<10DaPa change	-	56.7	-	64.9 (51.1–77.0)	49.1 (35.6–62.7)	0.14
	-ve equilibration	Raw data—daPa change	-3	-	0.58 (.48–68)	-	-	-
		<5DaPa change	-	53.8	-	63.2 (49.3–75.5)	50.9 (37.2–64.3)	0.14
		<10DaPa change	-	66.3	-	75.4 (62.2–85.8)	38.6 (25.9–52.4)	0.14
	±400daPa	Number results <5DaPa change /4	-	-	0.60 (.49-.69)	-	-	-
	together	Number results <10DaPa change /4	-	-	0.64 (.54-.74)	-	-	-
		Any of 4 results <5DaPa change	-	77.9	-	80.7 (68.0–89.9)	38.6 (25.9–52.4)	0.19
		Any of 4 results <10DaPa change	-	70.2	-	87.7 (76.3–94.9)	29.8 (18.4–43.4)	0.18
Patient-reported	Valsalva	Count no open out of 3	-	-	0.61 (.51-.71)	-	-	-
		≥2 out of 3 +ve	-	45.2	-	56.1 (42.3–69.2)	61.4 (47.5–74.0)	0.18
	Dry Toynbee	Count no open out of 3	-	-	0.61 (.51-.71)	-	-	-
		≥2 out of 3 +ve	-	66.3	-	77.2 (64.1–87.2)	45.6 (32.3–59.3)	0.23
Observed TM	Valsalva	Count no open out of 3	-	-	0.68 (.58-.78)	-	-	-
		≥2 out of 3 +ve	-	47.1	-	61.4 (42.3–69.2)	61.4 (47.5–74.0)	0.18
	Dry Toynbee	Count no open out of 3	-	-	0.67 (.58-.77)	-	-	-
		≥2 out of 3 +ve	-	61.5	-	73.7 (60.3–84.4)	54.4 (40.6–67.6)	0.28
TTAG	Valsalva	Count no open out of 3	-	-	0.59 (.49-.70)	-	-	-
		≥2 out of 3 +ve	-	41.3	-	49.1 (35.6–62.7)	70.2 (56.5–81.5)	0.19
	Dry Toynbee	Count no open out of 3	-	-	0.61 (.51-.71)	-	-	-
		≥2 out of 3 +ve	-	52.9	-	59.6 (45.8–72.4)	59.6 (45.8–72.4)	0.19
Continuous impedance	Valsalva	Count no open out of 3	-	-	0.61 (.51-.71)	-	-	-
		≥2 out of 3 +ve	-	37.5	-	43.9 (30.7–576)	71.9 (58.4–83.0)	0.16
	Dry Toynbee	Count no open out of 3	-	-	0.68 (.58-.77)	-	-	-
		≥2 out of 3 +ve	-	52.9	-	66.7 (52.9–785)	66.7 (52.9–78.5)	0.33
Sonotubometry	Dry swallow	Raw data–mean dB	5	-	0.41 (.31-.51)		-	-
		<0dB count out of 5	-	-	0.57 (.47-.68)	-	-	-
		<5dB count out of 5	-	-	0.58 (.47-.68)	-	-	-
		<10dB count out of 5	-	-	0.59 (.49-.70)	-	-	-
		3 out of 5 <0dB	-	37.5	-	45.6 (32.3–59.3)	70.2 (56.5–81.5)	0.16
		3 out of 5 <5dB	-	47.1	-	56.1 (42.3–69.2)	59.6 (45.8–72.4)	0.16
		3 out of 5 <10dB	-	58.7	-	66.7 (52.9–78.5)	47.4 (33.9–61.0)	0.14
Tubomanometry	300daPa	No open or R >1	-	56.7	-	68.4 (54.7–80.0)	59.6 (45.8–72.4)	0.28
	400daPa	No open or R >1		41.3		49.1 (35.6–62.7)	70.2 (56.5–81.5)	0.19
	500daPa	No open or R >1		39.4		47.4 (33.9–61.0)	71.9 (58.4–83.0)	0.19
	All 3 pressures	No open or R >1 out of 3	-	-	0.65 (.55-.75)	-	-	-
Tuboimpedance	300daPa	No open	-	26.9	-	36.8 (24.4–50.6)	84.2 (72.1–92.5)	0.21
	400daPa	No open	-	19.2		26.3 (15.5–39.6)	86.0 (74.2–93.7)	0.12
	500daPa	No open	-	7.7		12.3 (5.0–23.6)	96.5 (87.8–99.5)	0.09
	All 3 pressures	No opens out of 3	-	-	0.60 (.50-.70)	-	-	-
CETDA	Total score	Score	27	-	0.60 (.49-.69)	-	-	-
		Score ≥17	-	81.8	-	87.7 (76.3–94.9)	24.6 (14.1–37.7)	0.12
ETDQ-7	Total score	Score	25	-	0.59 (.48-.69)	-	-	-
		Score ≥15	-	71.0	-	80.7 (68.0–89.9)	24.6 (14.1–37.7)	0.05
ETS	Total score	Score	4	-	0.60 (.50-.70)	-	-	-
		Score ≤5	-	62.5	-	71.9 (58.4–83.0)	43.9 (30.7–57.6)	0.16
ETS7	Total score	Score	6		0.67 (.57-.77)	-	-	-
		Score ≤7	-	59.6	-	68.4 (54.7–80.0)	47.4 (33.9–61.0)	0.16
**Patulous ETD**						n = 12 PETD	n = 102 non-PETD	
Observed TM	Heavy breathing	+ve for change with breathing	-	4.8	-	33.3 (9.9–65.1)	99.0 (94.6–100.0)	0.32
TTAG	Heavy breathing	+ve for change with breathing	-	7.7	-	50.0 (21.0–78.9)	98.1 (93.0–99.8)	0.48
Impedance	Heavy breathing	+ve for change with breathing	-	6.7	-	50.0 (21.0–78.9)	99.0 (94.6–100.0)	0.49
Sonotubometry	Heavy breathing	+ve for change with breathing or ET remains open after swallowing	-	1.9	-	8.3 (0.2–38.4)	99.0 (94.6–100.0)	0.07
Tubomanometry	300daPa	R value < 0.2	-	5.8	-	33.3 (9.9–65.1)	98.1 (93.0–99.8)	0.31
	400daPa	R value < 0.2	-	4.8	-	33.3 (9.9–65.1)	99.0 (94.6–100.0)	0.32
	500daPa	R value < 0.2	-	5.8	-	25.0 (5.4–57.1)	97.1 (91.6–99.4)	0.22

Expert panel as the reference standard. CETDA = Cambridge Eustachian Tube Dysfunction Assessment, ETDQ-7 = Eustachian Tube Dysfunction Questionnaire 7,ETS(7) = Eustachian Tube Score (7), TTAG = tubo-tympanic aerodynamic graphy, TM = tympanic membrane

Development of a diagnostic model using multiple tests: The potential value of combining multiple tests was explored. Using penalised maximum likelihood estimation logistic regression, multivariable models were developed for predicting the likelihood of OETD. The LASSO (least absolute shrinkage and selection operator) penalty term was used[16]. The strategy was to fit models to a fixed set of predefined tests, and the LASSO approach may shrink some coefficients to zero. Thus, the approach provided a form of variable selection in addition to correcting for optimism and preventing very extreme predictions[17]. Two models were designed containing tests grouped by current clinical application. In one model (Research model), both routine tests and function tests that are infrequently used were included. In a second, simpler model (Clinical model), only tests that are routinely available in clinical practice were included. Diagnostic thresholds were not applied to continuous variables.

Tympanic pressure was considered by the clinical panel in making the final diagnosis of OETD, leading to potential for incorporation bias. Therefore, in sensitivity analyses this variable was excluded from some models, to explore whether the diagnostic value of the models was mainly driven by middle ear pressure.

Assessment of diagnostic model performance and internal validation: Model performance was assessed in terms of discrimination and calibration. Discrimination is the ability of the model to distinguish a patient with the endpoint OETD from a patient without OETD (with PETD or alternative diagnosis). The *c*-statistic for evaluating the discriminatory ability of a logistic regression model is equivalent to the ROC AUC. A *c*-statistic of 1.0 indicates perfect discrimination while a *c*-statistic of 0.5 indicates random discrimination[18]. For internal validation of the model discrimination, bootstrapping with 200 samples was used to obtain optimism-adjusted estimates of model performance statistics[18–20]. Calibration, the agreement between observed endpoints and predictions[21] was also assessed, with methodology for this and internal validation in the Supporting Information (S1 File).

#### Test accuracy based on latent class analysis

Latent class analysis (LCA) was used to combine multiple test results in order to estimate the proportion of patients with OETD, and the sensitivity and specificity of each of the index tests included in a 2-class latent class model. For LCA to be valid, the tests included in the analysis should be independent, measuring ETD in a distinct manner. Therefore, one test was selected for each aspect of ETD function (Table 1, column 5,), except where the test was considered sufficiently distinct (e.g. sonotubometry and impedance Toynbee for active opening).

When diagnosing ETD, there is no clear incentive to prioritise either sensitivity or specificity over the other. Therefore, for each test, the diagnostic threshold with the highest Youden’s index was selected for dichotomising ordinal and continuous test results, and LCA models were fitted. Four models were fitted: *All-test model*, with the panel diagnosis included as an index test alongside the tests of ET opening and PROMs; *No-panel model*, the all-test model without the panel diagnosis; *No-PROM model*, the All-test model without the PROM, and the *Open-test model* only including tests that measure ET opening. Each model containing a PROM included either the CETDA or ETDQ-7 (model variant A and B respectively). In additional variations of the models, tympanic pressure was excluded, since results from this test were used by the panel in making the final diagnosis for each patient.

LCA and regression analyses were performed using Stata 15 (StataCorp LP, College Station, Texas, USA).

#### Assessment of fatigue during repeated testing

Patients were required to repeatedly perform manoeuvres during assessment with TTAG, impedance and sonotubometry tests. To assess for evidence of fatigue and reducing ability, the nasopharyngeal pressures and results across the required 3–5 repetitions were compared using two-tailed AVOVA.

## Results

Data were obtained from 116 patients. Patient characteristics are outlined in Table 3. Six of the 116 patients were incorporated in analysis but had one missing test result due to: sonotubometry contraindicated with pacemaker (n = 1); erratic impedance baseline (n = 2); and erratic sonotubometry baseline (n = 3). An additional five patients were recruited but were later excluded (one withdrew consent during testing, three were unable to perform tubomanometry/tuboimpedance, and one could not complete the PROMs). One patient aspirated water during tubomanometry but quickly recovered, no other adverse events were recorded.

**Table 3:** Demographic and clinical characteristics of 116 participants.

Characteristic	No. (%)		Mean ±SD	
Age, years			50 ±16.3	
Male	50 (43)			
	**Test ear**	**Contralateral ear**	**Test ear**	**Contralateral ear**
Right ear	59 (48)	-		
Otoscopy normal	96 (83)	86 (76)		
Tympanogram type				
Type A	101 (87)	95 (82)		
Type B	0*	15 (13)		
Type C	15 (13)	6 (5)		
Middle ear pressure, daPa			-36.0 ±73.0	-46.9 ±50.2
PTA*, dB			26.8 ±19.6	—
PTA** >20dB	61 (54)	—		
Any symptoms present	113 (97)	69 (59)		

PTA = pure tone audiogram, SD = standard deviation

*type B tympanogram ears were excluded as not compatible with some tests

**average pure-tone hearing threshold levels at 500, 1000, 2000 and 4000Hz

### Expert panel diagnosis

**Fig 2:**
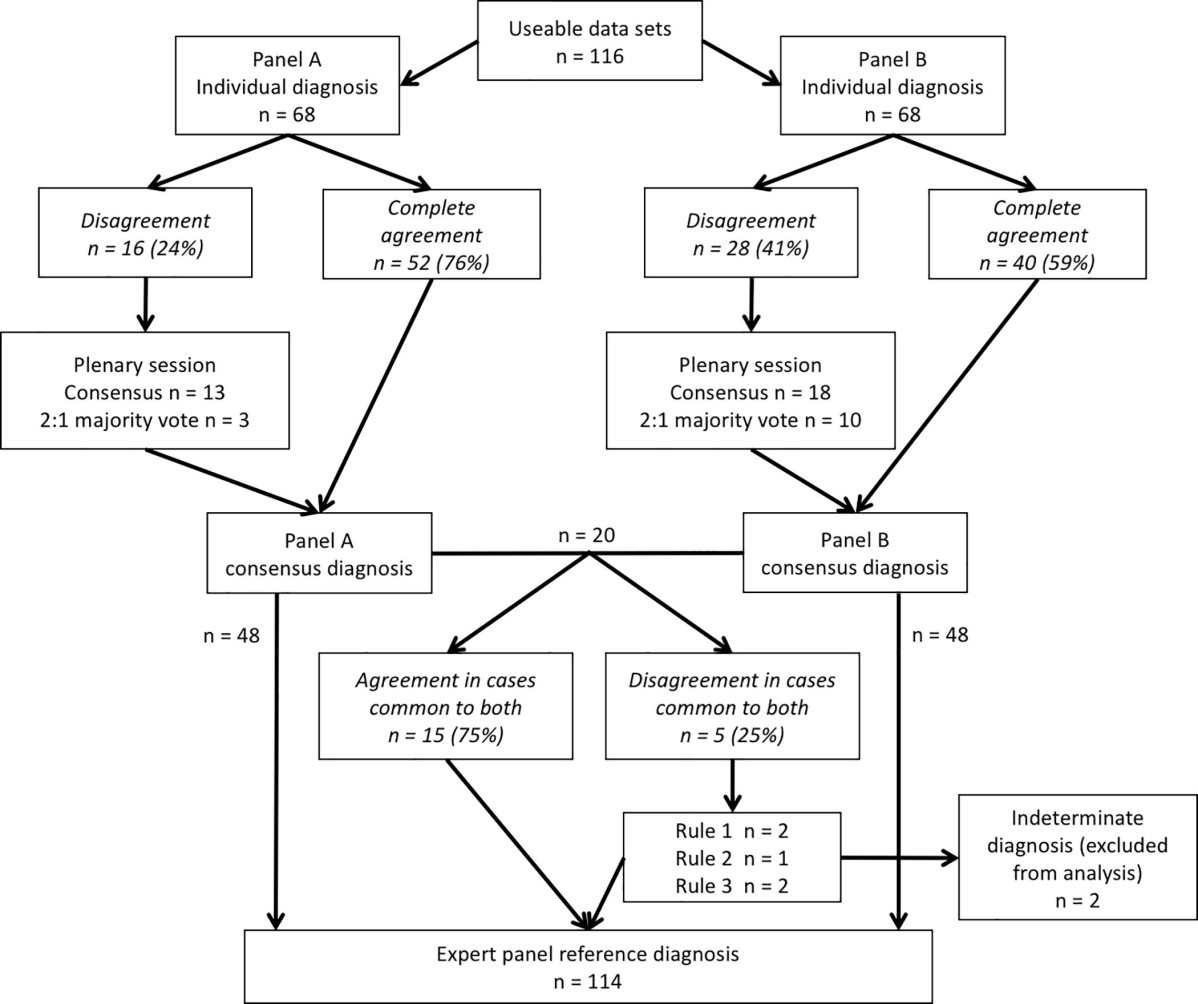
Flow chart for the expert panel diagnostic process. Rules were set to assign a diagnosis in the event of disagreement between panels A&B: 1. Diagnoses made via individual agreement were assigned over those with initial disagreement; 2. Diagnoses made at discussion via consensus were assigned over those made by a 2:1 vote; 3. If rule 1 or 2 could not be applied, the diagnosis was recorded as indeterminate.

Panel diagnoses were assigned as shown in Fig 2. Of the 116 patients, 57 (49.1%) had OETD, 12 (10.3%) had PETD and 45 (38.8%) had alternate diagnoses. For the remaining two (1.7%) patients, the panels could not agree and so the diagnosis for these patients was classified as indeterminate and they were excluded from further analysis. The proportion of cases assigned each primary diagnosis was similar between panels. In the 20 cases reviewed by both panels the diagnoses agreed in 15 individuals (75%), with Cohen’s κ = 0.55 (95% CI 0.21–0.89), representing a moderate strength of agreement[22].

### Patterns and correlations

Principle component analysis suggested the presence of four significant latent components (Eigenvalue>1) underlying the results of the OETD tests of ET opening, PROMs and panel diagnosis. All tests of ET opening weighted positively on the first component (which accounted for the greatest variance across all test results), while symptoms had a weak negative weighting on this component (Table 4). This pattern suggested that the first component represented ET function in terms of tube opening, as expected with the dataset mainly formed from tests of ET opening. The tests loading strongly for component 2 were the two PROMs and the panel diagnosis, while the tests of ET opening loaded weakly or negatively for this component; this component therefore appeared to relate to symptoms. The similar loading for the PROMs and panel diagnosis is not surprising, as symptoms are likely to have been a strong driving factor in the panel diagnosis. However, the loading patterns for components 1 and 2 suggest that symptoms and ET opening are distinct aspects of ETD which vary independently.

**Table 4:** Principle component analysis matrix.

Aspect of OETD	Test and parameter	Component			
		1	2	3	4
Passive opening	Observed Valsalva *Count/3*	0.79	-0.09	0.34	0.20
	Reported Valsalva *Count/3*	0.65	-0.07	0.32	0.37
	TTAG Valsalva *Count/3*	0.61	-0.31	0.60	0.01
	Impedance Valsalva *Count/3*	0.64	-0.15	0.50	-0.23
Active opening	Observed Toynbee *Count/3*	0.73	0.10	*-0*.*15*	0.32
	Reported Toynbee *Count/3*	0.61	0.13	*-0*.*32*	0.53
	TTAG Toynbee *Count/3*	0.66	-0.09	*-0*.*23*	0.01
	Impedance Toynbee *Count/3*	0.77	0.12	*-0*.*24*	-0.24
	Sonotubometry *<5dB count/5*	0.62	0.11	*-0*.*32*	-0.01
Both forms of opening	Tubomanometry 300 & 400daPa* R>1 or no open count/2	0.60	0.22	*-0*.*11*	-0.50
	Tuboimpedance *no-open count/3*	0.61	0.17	*-0*.*04*	-0.52
Pressure equalisation	Nine step *ME change <10daPa count/4*	0.50	0.23	*-0*.*43*	0.00
	Tympanometry *ME pressure daPa*	0.44	*-0*.*33*	*-0*.*04*	0.27
Symptoms	CETDA *Total score*	*-0*.*07*	0.89	0.32	0.13
	ETDQ-7 *Total score*	*-0*.*15*	0.91	0.26	0.02
All	Expert panel diagnosis *OETD*, *PETD or alternate diagnosis*	0.17	0.53	0.02	0.21

Positive loading >0.3 is highlighted with grey shading. n = 114.

*500daPa excluded as test performed poorly

For component 3, all tests measuring only passive ET opening had positive loading, while those measuring active and mixed opening all had negative loading. This suggests that passive and active ET dysfunction may be distinct, with some individuals expressing primarily passive or active dysfunction on tests of ET opening. Passive ET opening appeared more related to ETD symptoms than active opening on the basis of the loading for component 3. Loading for component 4 was overall weak with no clear pattern relating it to an attribute of ETD. PCA was not suitable to analyse PETD test results given the small PETD sample size.

In the OETD correlation matrix incorporating OETD tests of ET opening, PROMs and the panel diagnosis (S2 Table), most tests of ET opening correlated with one another, with only 4 of 78 pairings demonstrating *p*≥0.05. All opening test pairs with differences in results that showed a *p*≥0.01 comprised tests that measured different aspects of ET function (e.g. active and passive opening), further suggesting that some patients may have predominantly active or passive ET dysfunction. The pairwise correlation between PROM scores and tests of ET opening was insignificant (*p*≥0.05) in almost all cases, demonstrating a limited relationship between a patient’s symptoms and ET opening, as seen in the PCA. Also as suggested by the PCA, in the matrix the panel diagnosis was found to be correlated with PROM scores and not significantly correlated with most tests of ET opening. The lack of correlation between the panel diagnosis and tympanometry suggested that panel knowledge of tympanometry results had not led to significant incorporation bias: an important finding when including the test in the statistical models.

In contrast to OETD, the PETD correlation matrix (S3 Table) showed a different pattern, with the PETD panel diagnosis significantly correlated with the PETD tests of ET opening, and less strongly correlated with the PROMs. Again, the PROMs and tests of ET opening did not correlate.

### Test accuracy with panel diagnosis as reference standard

Table 2 presents estimates of test accuracy from all obstructive and patulous ETD tests using expert panel diagnosis as the reference standard. Diagnostic performance measured by the AUC and Youden’s index was in many cases poor: no single test had sensitivity and specificity >65% or *J*>0.5. Some tests had high sensitivity or specificity, depending on the threshold applied. Tests for PETD performed better than those for OETD, with higher specificity. ETS and ETS-7 scores were not assessed beyond this stage as they are composite scores incorporating the results of other index tests.

The low number of PETD cases (12 panel-diagnosed cases) prevented further analysis of the diagnostic accuracy of tests for PETD using LCA or regression modelling.

#### Selection of tests for inclusion in LCA and logistic regression models

For the regression analyses, all nine OETD tests of ET opening were included with the CETDA or ETDQ-7 in the Research model (Table 5), following selection of the test variant and threshold with the highest Youden’s index (based on results in Table 2). For LCA the same process was adopted to identify suitable variables, although some tests were excluded to ensure that those incorporated in the model were distinct, and not prone to similar test errors. To do this, a judgement was made on the basis of the underlying principle and methodology of each test. Selected tests were then grouped according to the four previously defined models (Table 5). For sonotubometry, a threshold of 5 or 10dB was not clearly superior, and so accuracy at each of the two thresholds was initially assessed in regression and LCA models. For tympanometry, thresholds of -50 and -100daPa were explored, with -50daPa found to give a higher accuracy, and correlate more closely with other tests of ET opening.

**Table 5:** 

		Combined test models			Latent class models					
Aspect of ETD evaluated	Selected ET function test	Regression variable	Clinical model	Research model	Threshold for test positivity	All-test Model	No-panelModel		No-PROM Model	Open-test Model
Symptoms*	CETDA score	Total score	**✓** (a)	**✓** (a)	Score ≥17	**✓** (a)	**✓** (a)			
	ETDQ7 score	Total score	**✓** (b)	**✓** (b)	Score ≥15	**✓** (b)	**✓** (b)			
ME pressure equalisation	Nine step test Tympanometry	+400 change in daPa ME pressure in daPa	**✓**	**✓** **✓**	+400 change <10daPa <-50daPa	**✓** **✓**	**✓** **✓**	**✓** **✓**		**✓** **✓**
Passive opening	Observed Valsalva Patient-reported Valsalva TTAG Valsalva	No. opening out of 3 No. opening out of 3 No. opening out of 3	**✓** ✘	**✓** ✘ ✘	2 out of 3 no open 2 out of 3 no open 2 out of 3 no open	**✓**	**✓**	**✓**		**✓**
Active opening	Sonotubometry Impedance Toynbee Observed Toynbee Patient-reported Toynbee	No. opening out of 5 (>5dB) No. opening out of 3 No. opening out of 3 No. opening out of 3	**✓** **✓**	✘ **✓**	3 out of 5 <5dB 2 out of 3 no open 2 out of 3 no open 2 out of 3 no open	**✓** ✘	**✓** ✘	**✓** ✘		**✓** ✘
Passive & active opening	Tubomanometry Tuboimpedance	No open or R>1 @30mbar No open @30mbar		**✓** ✘	No open or R>1 @300daPa No open @300daPa	**✓**	**✓**	**✓**		**✓**
All	Expert panel	NA	NA	NA	Obstructive ETD diagnosis	**✓**		**✓**		

*CETDA and ETDQ-7 included in separate models: model variant ‘a’ includes CETDA score and ‘b’ includes ETDQ7 score: the similarity of the PROMs precluded inclusion alongside one another in the models.

**✓** indicates tests selected for inclusion in the regression models and latent class analyses. Regression and LCA models evolved as tests were removed during analysis due to poor performance:

✘ indicates that the index test was removed during analysis. ME = Middle Ear, TTAG = Tubo-tympano-aerodynamic-graphy

#### Multivariable diagnostic models

While using the expert panel as the reference standard, the benefit of combining test results was explored. The outputs from the Clinical and Research models are shown in Table 6. Patient-reported Valsalva, TTAG Valsalva, sonotubometry (both 5 and 10dB thresholds) and tuboimpedance were de-selected in one or both of the models due to shrinkage of their coefficients to zero.

**Table 6:** The coefficients (= log odds ratio) can be used to calculate a patient’s risk of having OETD. As continuous, raw data were used, tympanogram middle ear pressure and nine step pressure change have a negative predictive effect.

		**A** (CETDA)			**B** (ETDQ-7)	
**Clinical Model**	**OR**	**95% CI**	**Coefficient**	**OR**	**95% CI**	**Coefficient**
PROM (A or B) *(per point)*	1.05	0.99–1.10	0.05	1.03	0.98–1.07	0.03
Tympanometry (*per dPa)*	0.99	0.98–1.00	-0.01	0.99	0.98–1.00	-0.01
Observed Valsalva *(per x/3)*	2.61	1.00–6.82	0.96	2.64	1.02–6.82	0.97
Observed Toynbee *(per x/3)*	1.21	0.40–3.67	0.19	1.25	0.42–3.76	0.23
Reported Toynbee *(per x/3)*	1.68	0.60–4.71	0.52	1.67	0.60–4.63	0.51
*Constant*			-2.40			-1.85
		**A** (CETDA)			**B** (ETDQ-7)	
**Research Model**	**OR**	**95% CI**	**Coefficient**	**OR**	**95% CI**	**Coefficient**
PROM (A or B) *(per point)*	1.05	1.00–1.11	0.05	1.03	0.98–1.07	0.03
Nine step (*per dPa change)*	0.99	0.98–1.01	-0.01	0.99	0.98–1.01	-0.01
Tympanometry (*per dPa)*	0.99	0.98–1.00	-0.01	0.99	0.98–1.00	-0.01
Impedance Toynbee *(per x/3)*	2.30	0.89–5.96	0.83	2.25	0.88–5.75	0.81
Observed Valsalva *(per x/3)*	2.21	0.89–5.44	0.79	2.29	0.94–5.60	0.83
Tubomanometry *(Y/N)*	1.76	0.71–4.37	0.57	1.72	0.70–4.23	0.54
*Constant*			-2.77			-2.14

OR = odds ratio.

1) Discriminatory ability of multivariable diagnostic models. The *c*-statistic was superior for both the Clinical and Research models (Table 7, ROC curves in S1 Fig) when compared to equivalent AUC values for the individual index tests (Table 2). The choice of PROM included (CETDA or ETDQ-7) had little effect on the performance of the models. The addition of more complex tests mainly used in research did not markedly improve accuracy above that seen when only tests used in standard clinical practice were included (optimism corrected *c*-statistic was 0.74 for clinical models and 0.76–0.77 for research models).

**Table 7:** Discriminatory ability of diagnostic models for OETD.

Model	*c*-statistic	95% CI	Optimism corrected *c*-statistic	Calibration slope
**Clinical A**	0.76	0.67–0.84	0.74 (SD 0.02)	0.83 (SD 0.18)
**Clinical B**	0.75	0.66–0.83	0.74 (SD 0.02)	0.79 (SD 0.19)
**Research A**	0.79	0.70–0.86	0.77 (SD 0.02)	0.80 (SD 0.18)
**Research B**	0.79	0.70–0.86	0.76 (SD 0.02)	0.79 (SD 0.19)

2) Calibration of multivariable diagnostic models. The calibration slope was between 0.79 and 0.83 for all regression models, as expected with relatively small datasets. This finding suggests that predictions made by the models regarding the probability of OETD are likely to be too extreme: low predictions too low, and high predictions too high[17] (calibration plots in S2 Fig).

### Latent class models

LCA provided an opportunity to assess the expert panel diagnosis as an index test along with other index tests. Results from the four LCA models are shown in Table 8i-iii. The proportion of cases assigned to the OETD class was 31.6–32.5% in models without the panel diagnosis, and 38.5–40.2% in models with the panel diagnosis. Because the latent variable (the OETD diagnosis) is unobserved it was not possible to determine if it was the same cases classified as OETD in the models with similar results, but this is the assumption given the stability of the findings[23].

**Table 8:** All test latent class model: the panel diagnosis included as an index test alongside the tests of ET opening and PROMs.

**Aspect of ET function evaluated**	**Test**	**All–Test Model a**					**All test Model b**				
		**Sens**	**95%CI**	**Spec**	**95% CI**	***J***	**Sens**	**95% CI**	**Spec**	**95% CI**	***J***
Symptoms	CETDA score	**87.6**	72.1–95.0	**22.4**	13.4–35.1	0.10	**—**	**—**	**—**	**—**	**—**
	ETDQ-7 score	**—**	**—**	**—**	**—**	**—**	**75.3**	58.7–86.8	**20.2**	11.9–32.2	-0.05
ME pressure equalisation	Tympanometry	**52.4**	27.4–76.2	**100.0**	0.0–100.0	0.52*	**54.6**	30.7–76.6	**100.0**	0.0–100.0	0.55*
	Nine step test	**62.4**	43.3–78.2	**81.8**	65.2–91.5	0.44	**61.7**	42.6–77.8	**80.2**	66.1–89.4	0.42
Passive opening	Observed Valsalva	**74.4**	56.5–86.7	**73.8**	54.8–86.7	0.48	**75.6**	57.9–87.5	**73.2**	55.9–85.5	0.49
Active opening	Sonotubometry	**77.6**	55.1–90.7	**71.4**	55.8–83.2	0.49	**79.5**	58.0–91.6	**71.4**	56.5–82.7	0.51*
Passive & active opening	Tubomanometry	**84.9**	62.7–94.9	**66.1**	49.7–79.4	0.51*	**86.4**	65.6–95.5	**65.7**	50.3–78.4	0.52*
All	Expert Panel	**79.1**	60.3–90.4	**69.6**	51.9–82.9	0.49	**79.0**	60.1–90.4	**68.2**	52.8–80.4	0.47
Percentage of cases in	**Class**	**%**	**95% CI**				**%**	**95% CI**			
each class	No OETD	**59.8**	39.9–76.9				**61.5**	44.1–76.3			
	OETD	**40.2**	23.1–60.1				**38.5**	23.7–55.9			
**Aspect of ET function evaluated**	**Test**	**No-Panel Model a**					**No-Panel Model b**				
		**Sens**	**95%CI**	**Spec**	**95% CI**	***J***	**Sens**	**95% CI**	**Spec**	**95% CI**	***J***
Symptoms	CETDA score	**86.6**	66.3–95.5	**20.8**	12.7–32.1	0.07	**—**	**—**	**—**	**—**	**—**
	ETDQ-7 score	**—**	**—**	**—**	**—**	**—**	**72.1**	52.9–85.6	**19.2**	11.5–30.3	-0.09
ME pressure equalisation	Tympanometry	**61.2**	30.4–85.1-	**97.7**	76.6–99.8	0.59*	**60.6**	33.6–82.3	**97.2**	81.9–99.7	0.58*
	Nine step test	**69.4**	41.5–87.9	**79.7**	66.6–88.6	0.49	**67.7**	44.084.9-	**78.7**	65.9–87.7	0.46
Passive opening	Observed Valsalva	**76.1**	54.5–89.5	**68.7**	53.3–80.8	0.45	**76.9**	55.2–90.0	**68.8**	54.8–80.1	0.46
Active opening	Sonotubometry	**87.0**	58.2–97.0	**69.9**	53.5–82.4	0.57*	**89.2**	59.6–97.9	**70.7**	55.7–82.3	0.60*
Passive & active opening	Tubomanometry	**90.9**	67.4–97.9	**62.7**	46.2–76.7	0.54*	**92.0**	68.7–98.4	**63.0**	47.9–75.9	0.55*
All	Expert Panel	**—**	**—**	**—**	**—**	**—**	**—**	**—**	**—**	**—**	**—**
Percentage of cases in	**Class**	**%**	**95% CI**				**%**	**95% CI**			
each class	No OETD	**68.1**	47.4–83.5				**68.3**	50.9–81.8			
	OETD	**31.9**	16.4–52.5				**31.6**	18.2–49.0			
**Aspect of ET function evaluated**	**Test**	**No-PROM Model**					**Open-Test Model**				
		**Sens**	**95%CI**	**Spec**	**95% CI**	***J***	**Sens**	**95% CI**	**Spec**	**95% CI**	***J***
Symptoms	CETDA score	**—**	**—**	**—**	**—**	**—**	**—**	**—**	**—**	**—**	**—**
	ETDQ-7 score	**—**	**—**	**—**	**—**	**—**	**—**	**—**	**—**	**—**	**—**
ME pressure equalisation	Tympanometry	**54.1**	29.8–76.5	**100.0**	0.0–100.0	0.54*	**60.0**	32.5–82.3	**97.6**	79.5–99.8	0.58*
	Nine step test	**62.0**	42.9–78.0	**80.6**	66.2–89.9	0.43	**68.0**	43.5–85.4	**79.4**	66.5–88.2	0.47
Passive opening	Observed Valsalva	**75.2**	57.5–87.2	**73.3**	55.6–85.7	0.49	**76.2**	55.0–89.3	**69.1**	54.4–80.7	0.45
Active opening	Sonotubometry	**78.9**	57.3–91.3	**71.3**	56.3–82.8	0.50	**87.7**	59.5–97.2	**70.7**	55.2–82.5	0.58*
Passive & active opening	Tubomanometry	**86.2**	65.1–95.4	**65.9**	50.2–78.8	0.52*	**91.3**	68.4–98.1	**63.3**	47.6–76.6	0.55*
All	Expert Panel	**79.0**	60.0–90.4	**68.5**	52.7–80.9	0.48	**—**	**—**	**—**	**—**	**—**
Percentage of cases in	**Class**	**%**	**95% CI**				**%**	**95% CI**			
each class	No OETD	**61.0**	43.1–76.5				**67.6**	49.1–81.8			
	OETD	**39.0**	23.5–56.9				**32.4**	18.1–50.1			

‘a’ model includes the CETDA whereas ‘b’ model includes the ETDQ-7. No-panel latent class model: the All-test model without the panel diagnosis. No-PROM latent class model: the All-test model without the PROM). Open-test latent class model: only tests that measure ET opening included. Sens = sensitivity, Spec = specificity, *J* = Youden’s index (*J*) (* if >0.5),— = Not included, Impedance Toynbee (not shown) was excluded during analysis.

The addition of the panel diagnosis to the simpler models had a consistent effect: reducing the sensitivity and specificity of all other index tests by classifying a greater proportion of the cohort as OETD cases. In contrast, the addition of either the CETDA or ETDQ-7 to the models had little impact on class allocation, with the models suggesting the PROMs had no diagnostic value, *J* = -0.09 to 0.10. All models, with and without the panel diagnosis, demonstrated a consistent finding that tympanometry, sonotubometry and tubomanometry (using 300daPa) provided the greatest diagnostic value, being the only tests with *J* >0.5. Tests measuring ME pressure equalisation tended to have had higher specificity than tests measuring other aspects of ET opening.

Tympanometry was excluded from early models (not shown) due to concern over incorporation bias, but the effect on the model of including tympanometry seemed similar to the effect of other tests of ET opening, and unlike the effect of including the panel diagnosis. Sonotubometry interpreted with a 10dB threshold was included in some early LCA models, but was excluded due to model instability, unlike when sonotubometry was included at a 5dB threshold.

#### Fatigue

There was no evidence of a systematic change in patient manoeuvre ability (measured by generated nasopharyngeal pressure) or test results across the immediate test repeats (ANOVA p value range 0.40–0.98) (S4 Table).

#### Patient and assessor burden

All tests could be completed in a sufficiently short period of time by a single trained individual for them to be practical for use in routine clinics. The mean time taken to perform all objective tests together was 40 min 21 sec (SD 283 sec), in addition to the time for PROM completion (not timed). The recorded time to complete each test included patient instruction, attempts to gain competence or useable data (if required), and data recording (Table 9). Tests were well tolerated overall, with difficulty and discomfort ratings highest for tubomanometry and tuboimpedance (Table 9), reported by patients to be due to the applied sudden-onset nasopharyngeal pressure during these tests.

**Table 9:** Time to complete each test and patient-reported difficulty/discomfort.

			Reported by patient	
ET Opening Test	Repetitions	Time to complete Mean (SD)	Difficulty /10 Median (range)	Discomfort /10 Median (range)
Tympanometry	1	1m 4s (34s)	1 (1–2)	1 (1–3)
Nine-step test	1	6m 28s (82s)	1 (1–5)	1 (1–7)
Patient-report & TM observation	6+1*	3m 3s (51s)	1 (1–6)	2 (1–8)
TTAG	6+1*	5m 37s (78s)	1 (1–4)	2 (1–9)
Impedance	6+1*	6m 3s (85s)	2 (1–6)	1 (1–6)
Sonotubometry	5+1*	3m 53s (50s)	1 (1–6)	1 (1–6)
Tubomanometry	3	7m 53s (138s)	3 (1–10)	3 (1–8)
Tuboimpedance	3	6m 35s (143s)	3 (1–10)	2 (1–8)

Values for patient-report and tympanic membrane (TM) observation were recorded together. TTAG = Tubo-tympano-aerodynamic-graphy.

* = additional PETD test for heavy breathing performed with OETD tests

## Discussion

Eustachian tube dysfunction is poorly defined and often presents a diagnostic challenge to clinicians. OETD or PETD diagnoses have often been applied to patients (or individual ears) based on clinical assessment relying on symptoms, otoscopy, tympanometry and pure tone audiometry. Although largely unmeasured, the assumption has been that the clinical diagnosis reliably translates to the ability of the ET to perform its physiological functions; opening to permit ME pressure equalisation and drainage, and protection of the middle ear. In recent years this link has been questioned, with reports that symptoms and the results of objective tests (tympanometry[24], tubomanometry[25, 26] and middle ear pressure equilibration tests[27]) were not closely matched. In this study PCA and correlation matrices demonstrated that patient symptoms measured by PROMs, and ET function measured by tests of ET opening for OETD and PETD, appear poorly correlated and vary independently. The panel diagnosis, as a close correlate of current clinical diagnosis, was found to be closely related to OETD symptoms, but not the measured underlying ET function.

### Selecting an ideal reference standard for OETD

Two approaches for establishing test accuracy were explored. A consistent finding was that the tested PROMs had limited or no value as a diagnostic tool for OETD, highlighted by the very low Youden’s index and lack of influence on the latent class model output. While the PROMs consistently showed good sensitivity, the 71.0% to 81.8% overall PROM positive rate in the cohort resulted in very poor specificity. It is likely that the nature of the symptoms covered by the PROM items meant that patients with conditions such as hearing loss, Meniere’s Disease or temporomandibular joint (TMJ) dysfunction also scored highly, as has previously been demonstrated[11].

The clearest difference between using the panel diagnosis and LCA was the percentage of individuals diagnosed as OETD: approximately 49% according to the panel, and 32% according to LCA models without the panel (39% incorporating the panel). This difference had the effect of producing lower sensitivity values for the tests of ET opening when using the expert panel as the reference standard.

There are two possible reasons for the differences between reference diagnoses:

1. OETD was over-diagnosed clinically. Clinical diagnosis relies heavily on symptoms since examination and basic tests may both be normal. Symptoms are non-specific, as confirmed by the PROM performance, and so there may have been a high false positive rate in the panel diagnoses. Based on the PETD opening test results, clinical over-diagnosis of PETD may also have occurred.
2. Test of ET opening under-diagnose OETD. ET function may vary over time, and in some patients in whom OETD is intermittent the single testing episode may have occurred during a period of normal ET function, leading to a false negative. An example may be those patients who only suffer OETD during significant ambient pressure changes (baro-challenge). By re-testing individuals after an interval[10], on a separate day, or even under different conditions such as in a pressure chamber[28], it may be that intermittent OETD can be detected in more ears. Alternatively, the tests of ET opening may fail to diagnose those who only have a mild form of the disorder, perhaps because the obstructed ET still opens under the non-physiological conditions during many of the tests, or in the case of PETD, the ET opens and closes in a normal manner except during exercise or other provocation.

The expert panel consensus process demonstrated that even experienced clinicians could not agree on the diagnosis for certain patients, and together with the poor correlation with tests of ET opening, this suggests that the panel diagnosis reference standard may be imperfect. However, completely removing the clinical acumen of an experienced clinician from the diagnostic process for ETD risks the loss of a highly-sophisticated diagnostic ‘test’. Although the tests of ET opening were found to correlate with each other, and measure physiologically important aspects of ET function such as ME pressure equalisation, there are likely to be more aspects to normal ET function than can be objectively measured currently. The LCA No-PROM model (given the lack of diagnostic value from PROMs) therefore appears an attractive experimental means to combine clinical diagnosis with measured ET function.

### Clinical and research implications: Diagnosing and measuring OETD

This study suggests that current methods of clinical diagnosis alone (review of a patient’s history, examination, tympanometry and audiometry), even in the form of an expert panel’s consensus opinion, may be inadequate to diagnose OETD. The panel diagnosis analysed as an index test appeared equivalent or inferior to some tests of ET opening. As indicated by the components of the PCA, if the panel diagnosis and tests of ET opening both measure ETD, they measure different aspects of the condition, and so are complementary, rather than alternative diagnostic tools. While the PROMs were aligned with the panel diagnosis in the PCA, unlike the panel they were found not have diagnostic value due to poor specificity, and so analysis using the existing PROMs should be omitted from the diagnosis of ETD.

It appears therefore that there are two aspects of OETD that should be incorporated into clinical and research practice when diagnosing ETD: the clinical assessment, representing patient history, examination and symptoms (rationalised in a manner that the tested PROMs cannot to ensure adequate specificity), and objective measures of ET function such as the tests of ET opening. Current methods of diagnosis based on clinical assessment[1] may not select the cohort of patients who would benefit most from interventions which are often designed to aid ET opening. They also appear inadequate in situations where a higher certainty of diagnosis is required, such as when surgical interventions such as balloon Eustachian tuboplasty are being considered, or when conducting clinical research.

This study builds on previous work to identify the most useful ET function tests in terms of reliability and accuracy[9, 10]. Given the potential issues with the flawed nature of the expert panel reference it appears that the results from the latent class models are more reliable, where tympanometry, sonotubometry and tubomanometry had the highest Youden’s index and therefore greatest potential diagnostic value for OETD.

On the basis of our data using two current PROMs, PROMs should not be used in the diagnosis of ETD, though they may have a role in quantifying and documenting symptoms, either to aid in the selection of confirmed ETD patients where surgical intervention may be justified, or if shown to be responsive, to monitor the course of disease over time or following treatment. Current PROMs are designed to be self-completed by the subjects, which introduces variability in the interpretation of the questions. Future research could be done into the development of an instrument that would act as a framework for taking the medical history, inquiring about a standard set of symptoms relevant to ETD. If validated, such an instrument might improve the initial selection of patients for testing.

### Clinical and research implications: Diagnosing and measuring PETD

For PETD, only one reference standard was assessed as the sample size of affected cases (based on review of PETD tests of ET opening and the panel diagnosis) was deemed too small to allow reliable latent class analysis. Nonetheless the available data represent an advance in our understanding of tests for PETD, having investigated a number of tests in a single, clinically-relevant mixed cohort at the same point in time. The panel diagnosis and PETD tests of ET opening demonstrated significant correlation, suggesting that for PETD, the recognition of characteristic features in the history[29] may ensure that clinical diagnosis reflects the measured ET function better than is the case for OETD. A diagnostic feature of PETD is reported to be movement of the tympanic membrane synchronised with heavy breathing, and the continuous impedance and TTAG tests measuring this feature had the highest diagnostic accuracy in this study. Sonotubometry performed poorly using the tested protocol, however newer techniques may be superior[30].

### The role of test combinations

Previous work has suggested that combining different ET function tests may improve diagnostic accuracy for ETD[2, 31, 32]. This was found to be the case in the regression models, where combining either basic, currently-used tests (Clinical model) or combining basic and advanced tests (Research model) improved the accuracy over the individual measures. Interestingly the additional improvement in accuracy using the Research model over the Clinical model was negligible, but this may be due to the potentially flawed expert panel reference standard, which was shown to relate poorly to the tests of ET opening included in the Research model.

For the regression models to be clinically useful as a diagnostic tool, they must be interpreted via a score chart, nomogram or calculator[17] using the coefficients identified for each test, and applying the patient’s test results to generate a probability of the patient having OETD. Given the reference standard on which the model was built, it was felt that a calculator based on our model would have limited clinical utility. However, the modelling provides further evidence that the results of ET function tests should be combined, ideally mathematically.

### Recommended ETD diagnostic pathway for clinical and research use

**Fig 3:**
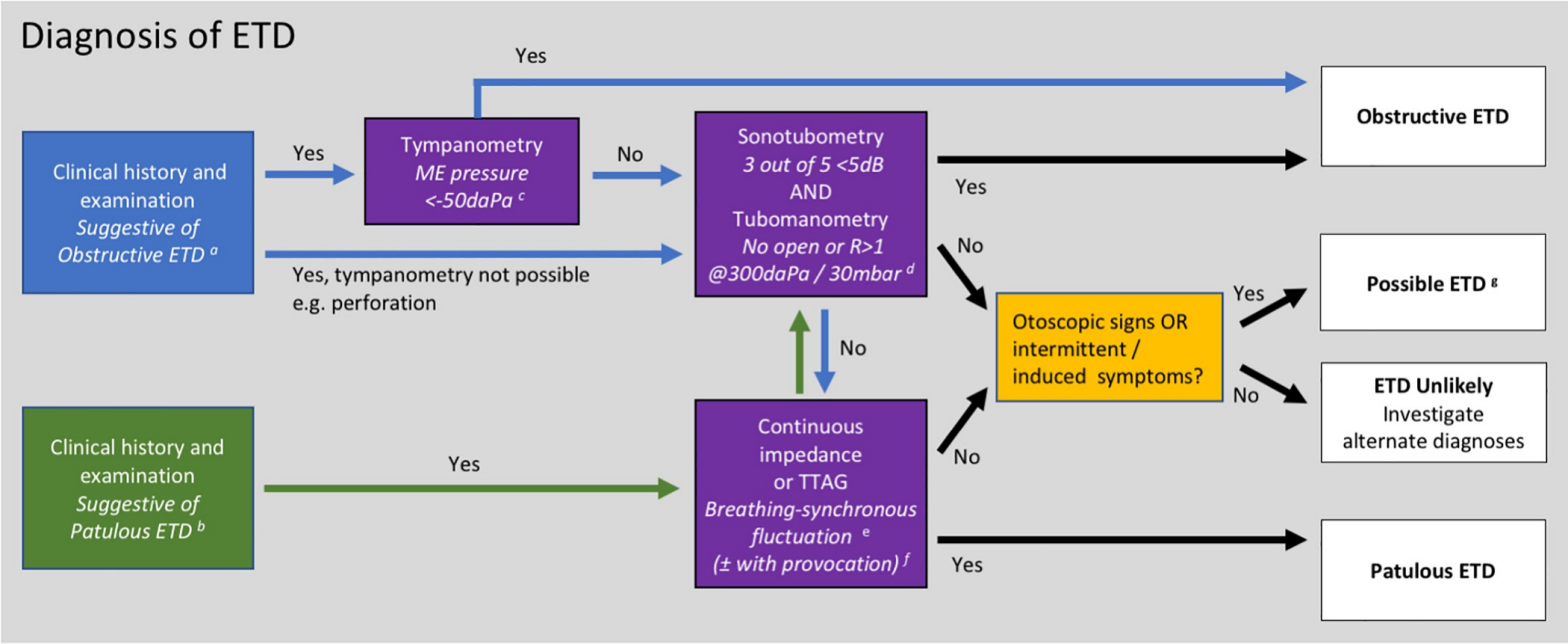
Proposed diagnostic pathway for ETD. ^a, b^ While clinical assessment of a patient’s history and conventional examination may not be diagnostic of ETD, they are nonetheless an important means to identify suitable patients for investigation. ^c^ Effort should be made during assessment of the clinical history to identify habitual sniffing, as a negative middle ear pressure in these individuals may not indicate OETD, and further testing should be undertaken. ^d^ Described diagnostic thresholds are based on the equipment and protocols used in our study, but may require adjustment if alternative methods are used ^**e**^ TTAG is recommended if a tympanic membrane perforation is present. ^f^ A simple provocation test for use in clinic is asking the patient to exercise (jog on the spot or climb a flight of stairs) prior to testing. ^g^ Consider repeating tests on a separate occasion to improve sensitivity in patients with variable ET function. Patients with baro-challenge induced OETD may present in this group.

On the basis of our findings, and a foundation of previously published work, we now have a much clearer understanding as to which tests of ET function should be employed, how these tests should be interpreted, and therefore, how ETD should be diagnosed. To guide clinicians and researchers, a diagnostic flow chart for ETD is proposed, incorporating the key assessment steps required to establish a diagnosis of OETD or PETD (Fig 3). The pathway incorporates tests selected on the basis of diagnostic performance, repeatability, feasibility and patient acceptability.

The pathway has been designed to enable the selection of patients for intervention, whether this be for clinical practice or research. For this reason, it has been designed to maximise specificity when selecting individuals with OETD or PETD, as both clinicians and patients often want a high level of certainty in the diagnosis before initiating an intervention.

#### Pathway stage 1: Clinical assessment

The pathway begins with clinical assessment, allowing the selection of patients suspected of having ETD, and initially targeting testing at the form of ETD thought to be most likely (OETD or PETD). Pre-selection of patients is standard practice when tests are applied in every field of medicine, ensuring that the tested population has a higher incidence of the target disorder, thereby reducing the cost and patient burden of unnecessary testing and improving the predictive value of the applied tests.

#### Pathway stage 2: ET function tests

In patients suspected of having OETD with an intact TM without effusion, tympanometry should be applied as the first test of ET function. This is recommended because tympanometry has very high specificity, close to 100% in latent class models, although sensitivity was poor. Therefore, if ME pressure measured by tympanometry is lower than -50daPa patients can be diagnosed as having OETD without further testing. One exception to this results from the predisposition to inappropriate (often habitual) sniffing of some patients with PETD[33]. Clinicians should be aware that if patients admit to regular sniffing, this may cause a transient negative ME pressure in those with intermittent PETD, and locking of the ET at negative pressure may even create a temporary OETD.

If tympanometry is normal, or the patient reports a history of inappropriate sniffing, further testing is warranted. Sonotubometry and tubomanometry demonstrated the best diagnostic performance for OETD, and while the two tests have good correlation, they do not diagnose exactly the same cohort. For these reasons, and to maximise diagnostic specificity, it is currently recommended that both tests should be positive if OETD is to be diagnosed with certainty.

While the use of tubomanometry and sonotubometry is encouraged, they are currently not available in many centres. If this is the case, observed Valsalva has the best diagnostic performance of the tests not requiring specialist equipment. This test does however have certain disadvantages, the main one being a high degree of variation between patients regarding the nasopharyngeal pressure generated, even following training, that has been shown to affect the rate of ET opening and therefore test outcome[9, 10]. Observed Valsalva also only tests passive ET opening, therefore potentially missing individuals with OETD due to failure of active ET opening. If the tympanic membrane is not intact, basic methods for objectively measuring ET function are currently not available.

For patients suspected of having PETD, the detection of ME pressure changes synchronised with nasopharyngeal pressure changes on breathing appears to be the best form of diagnostic test: although our own diagnostic study only incorporated a small sample size, the test is widely accepted, and the mechanism for positive test results with patency of the ET is clear. Impedance and TTAG methods of measurement are recommended for use in intact and perforated TMs respectively, as both tests were found to have comparable sensitivity, specificity and ease of use. If specialist equipment is not available, forced breathing with observation of the tympanic membrane provides a diagnostic option for PETD if the tympanic membrane is intact.

If patients have negative results from tests of ET opening on the side of the diagnostic pathway they started on (i.e. testing for obstructive or patulous forms of ETD), it is suggested that they cross over to undergo further testing for the other form of ETD. This step is suggested due to the possibility of initial incorrect targeting of testing, on the basis of non-specific symptoms or examination findings.

#### Pathway stage 3: Repeat testing

Patients with suspected ETD who have negative tests of ET opening for both OETD and PETD still cannot reliably be confirmed as non-ETD affected. The first reason for this is the intermittent nature of obstructive and patulous ETD in some individuals, as ET function may vary over even a relatively short period[10]. If clinical suspicion is high, repeat testing on another occasion is recommended in test-negative ears, to improve the likelihood that intermittent ETD is detected[34].

A second reason for an apparent absence of ETD during testing may be that the patient’s ETD only occurs under certain circumstances that cannot be replicated during testing, such as during baro-challenge. For PETD, it may be possible to replicate some circumstances that provoke symptoms at the time of testing, and the pathway includes a recommendation that exercise is used as a simple means of provocation[29].

#### Pathway stage 4: Current diagnostic uncertainty

The final stage involves an assessment as to why tests of ET opening may be negative. A diagnosis of *Possible ETD* is recommended for those patients who have symptom or findings suggestive of intermittent, or situation-specific ETD (e.g. arising on baro-challenge), that we cannot detect with existing diagnostic tools if the patient is not affected by ETD at the time of testing. If a patient’s symptoms are continuous and present at, or around, the time of testing, it appears reasonable to label these individuals as *ETD Unlikely*, particularly after testing on more than one occasion.

The Possible ETD group clearly may contain individuals with an alternate diagnosis. In the absence of additional diagnostic methods for ETD, it may be difficult to finally exclude or detect ETD, and the clinician should consider investigating patients in this group for alternate conditions. Care should be taken when treating patients diagnosed as Possible ETD, and it is not recommended that this group is recruited to early-stage interventional trials.

### Active and passive ET function

It is widely acknowledged that the ET may open actively (via muscular function) or passively (due to a pressure differential), and although a predominance of active or passive dysfunction is assumed in certain circumstances (e.g. cleft palate and rhinosinusitis respectively) the evidence of this distinction from objective testing has been limited[2, 35]. While the overall good correlation in this study between tests measuring only passive or active function suggests that in most cases the two vary together, a distinction between active and passive ET opening was supported by component 3 of the PCA. In future research, it would be valuable to measure active and passive dysfunction separately and determine if patients with a predominance of one or other dysfunction have a similar clinical course or response to interventions.

### Limitations of the study

Both methods of classifying the disease status of individuals have flaws, though this study represents an advance over previous diagnostic case-control studies that relied on a single imperfect diagnostic method. While expert consensus may be expected to provide a more accurate diagnosis than a single clinician, the experts in this study were handicapped by not being able to question or examine the patients themselves, instead relying on a detailed case summary. In their normal practice the members of the expert panel may have had access to more test results than those provided here (audiometry and tympanometry), and so the expert diagnoses may not fully represent the diagnostic ability of the panel members. The case summaries presented to the panels reflected the information collected in standard practice in most ENT units, thus ensuring blinding to the index test results to prevent incorporation bias. Expert panel members drew attention to the importance of inappropriate (sometimes termed habitual) sniffing in patients with PETD[33]. The presence or absence of this symptom was not recorded in the case histories, potentially reducing the validity of tympanometry interpretation. Although PETD opening tests suggest this was not seen in this study, some patients may have PETD and intermittent negative ME pressure, and the proposed diagnostic pathway has been designed to take account of this often-misdiagnosed group.

The primary concern regarding the latent class analysis was that although efforts were made to select index tests for inclusion that were independent, this condition could have been violated if tests were prone to similar errors, or incorporation bias was an issue due to panel knowledge of patients’ symptoms and tympanometry results. The latter appears not to have been a problem as the PROMs were found to be non-diagnostic, and tympanometry results did not correlate with the panel diagnosis.

Not all tests reported to measure or diagnose ETD could be included in this study. For more complex tests such as sonotubometry there are multiple methods described to perform and interpret the tests that were not explored in this study[30, 36, 37]. Numerous tests have been described that require a perforated tympanic membrane, and these often allow greater analysis of tubal opening and closing[2]. Assigning equivalence between the results of different tests in ears with intact and perforated tympanic membranes will require further work, possibly utilising tests such as sonotubometry which do not require a specific tympanic membrane state. This study also did not examine the role of endoscopic examination of the lumen of the cartilaginous ET. Although endoscopy during dynamic manoeuvres has not been reported to be a test of ET function, the findings may be of value in diagnosing the presence or cause of ETD, and differentiating between obstructive and patulous forms of the disease[38]. For those tests that were included, the findings should still be considered in the context of a relatively small sample size. The results of this study therefore provide direction for further research.

This study was designed to overcome the limited applicability of existing case-control studies by prospectively recruiting a clinically-relevant mixed cohort of individuals with highly varied forms and severity of presentation. The accuracy estimates from this study are therefore likely to be representative of patients at other institutions. However, to ensure viability of the study, in particular the ability to apply a wide range of tests to a single individual, certain exclusion criteria were applied, such as the need for an intact tympanic membrane and aerated ME. These requirements meant that diagnostic ability has not been assessed in all presentations of ETD, and ETD may be more severe or prevalent in ears with a perforated tympanic membrane or significant ME disease. Children also provide a separate population where the validity of extrapolating data from this study is unclear. The aetiology and natural course of ETD in children appears to differ from that in adults[39], and additional difficulty will arise in testing due to patient understanding and compliance. However, the recommended diagnostic process from this study provides a basis for further work in children.

## Conclusions

The findings in this study demonstrate the importance of measuring ET function in clinical practice. ETD should be diagnosed via clinical assessment combined with testing of ET opening specific to OETD and PETD, in contrast to previous recommendations that symptoms should be central to the diagnosis and definition[1]. The use of tests of ET opening may improve the selection of candidates for intervention, and allow the objective assessment of outcomes. Currently it is recommended that only patients with ETD diagnosed via positive tests of ET opening should be included in interventional trials. PROMs should not be used in the diagnostic process, but may have a role in characterising diagnosed cases and monitoring their response to treatment. Particular importance should now be given to reducing diagnostic uncertainty, and eliminating the need for a Possible ETD group, by developing practical ways to detect ears with intermittent or induced ETD.

## Supporting information

S1 Fig Discriminatory ability of the logistic regression models (ROC curve). (TIFF)

S2 Fig Calibration plots for the logistic regression models (ROC curve). (TIFF)

S1 Table Symptoms associated with ETD (both obstructive and patulous) obtained via literature review. (DOCX)

S2 Table OETD Test/PROM correlation matrix. Variables are the same as those recorded in Table 4. Bivarate Pearson correlation (r) shown, p value (two tailed) indicated by shading: dark grey shading p<0.01, and light grey shading p<0.05.
(DOCX)

S3 Table PETD Test/PROM correlation matrix. Variables are the same as those recorded in Table 4. Bivarate Pearson correlation (r) shown, p value (two tailed) indicated by shading: dark grey shading p<0.01, and light grey shading p<0.05.
(DOCX)

S4 Table Comparison of nasopharyngeal pressure and test outcomes across immediate repeats at a cohort level (x3 for all tests except sontotubometry which was repeated x5). If present, fatigue may be expected to affect either patient manoeuvre ability of test results. Continuous variables only assessed. Tubomanometry and Tuboimpedance were not assessed as differences at 30/40/50mbar are expected.
(DOCX)

S1 File Supporting information. (DOCX)
