# Supplementary figures and images for: Eustachian tube dysfunction: A diagnostic accuracy study and proposed diagnostic pathway
Source: PLoS One. 2018 Nov 8;13(11):e0206946. doi: 10.1371/journal.pone.0206946 (PMC6224095; doi:10.1371/journal.pone.0206946)

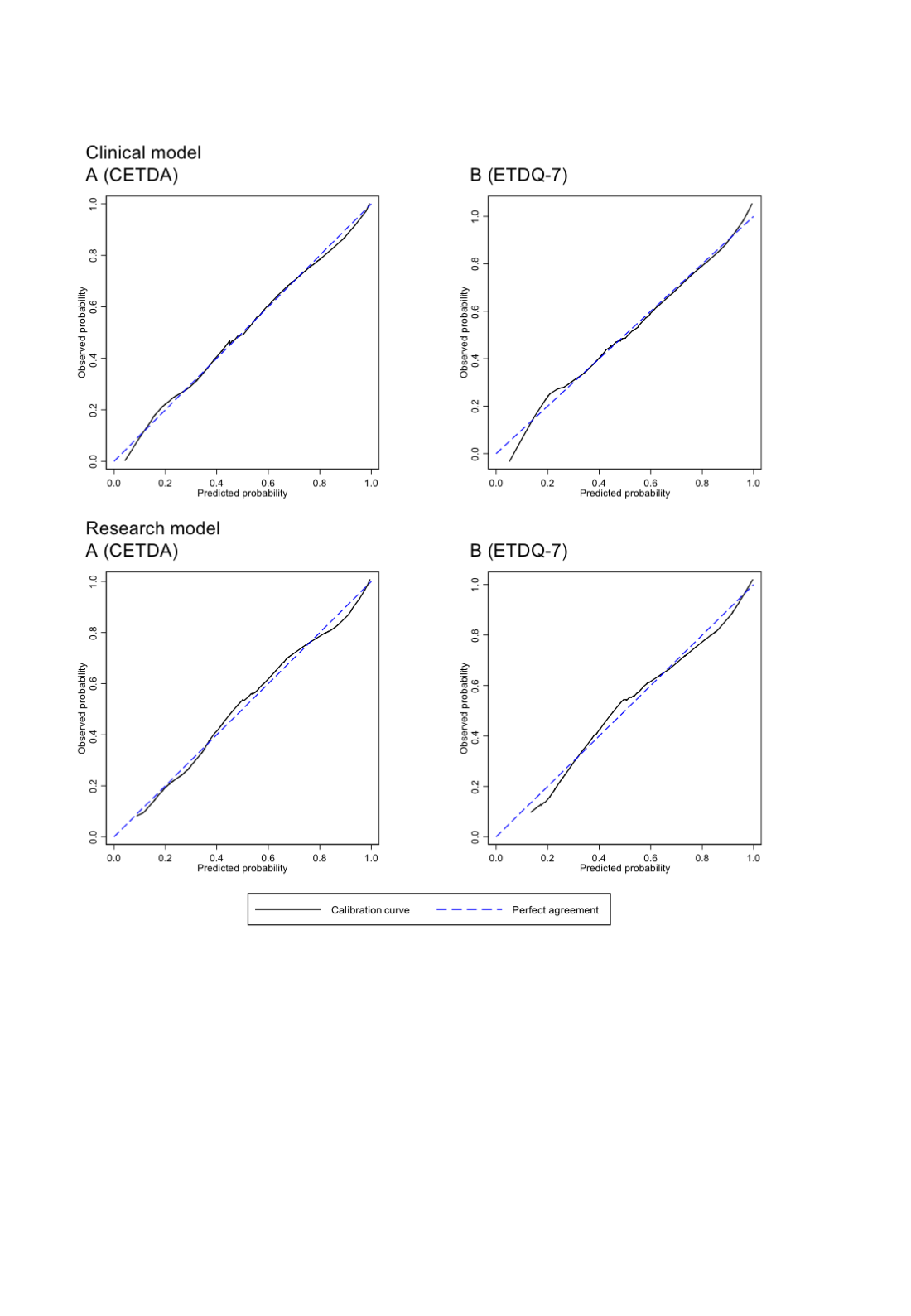

Supplement: S1 Fig — (TIFF) [file pone.0206946.s001.tiff]

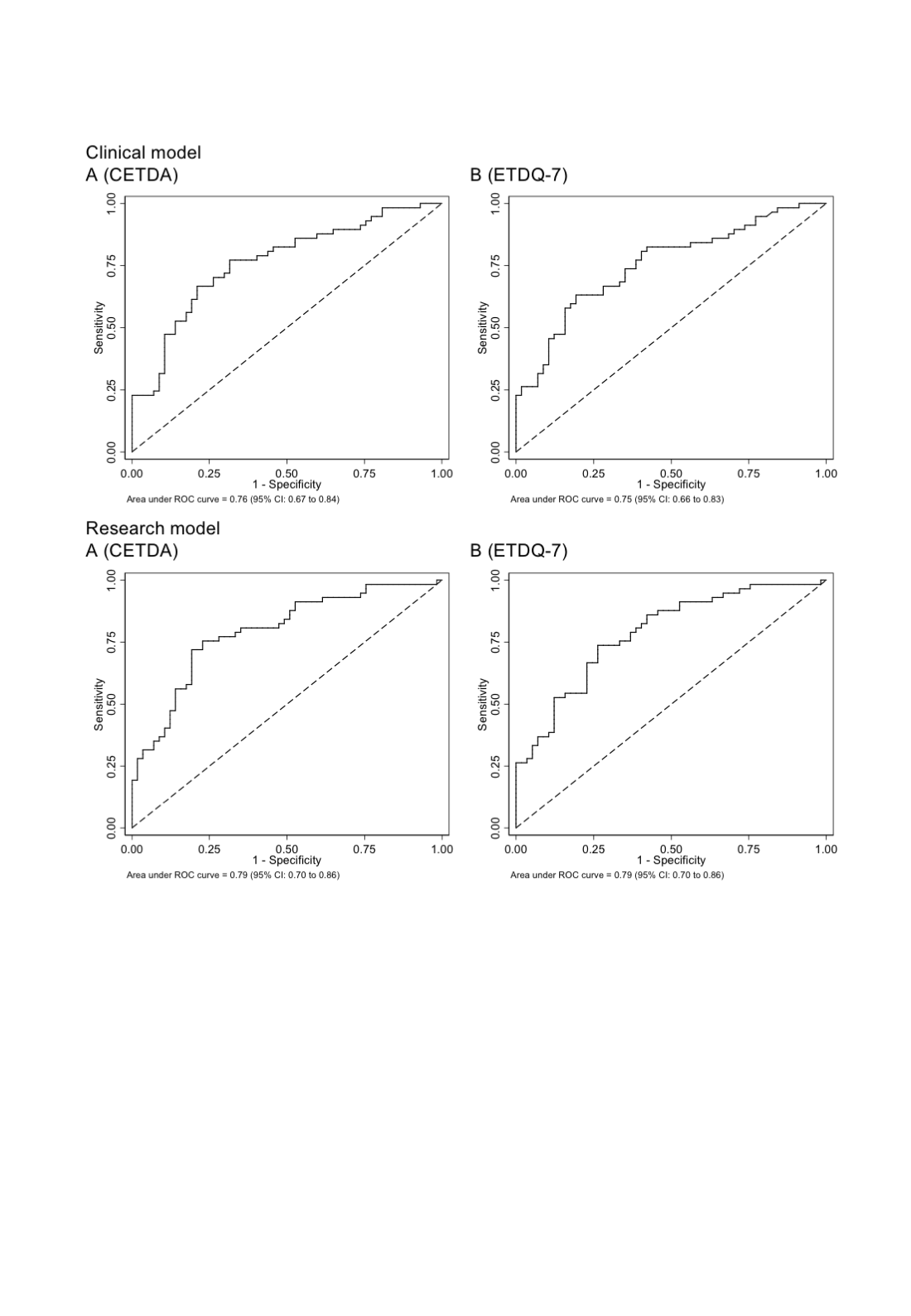

Supplement: S2 Fig — (TIFF) [file pone.0206946.s002.tiff]
