# Supplementary material for: Eustachian tube dysfunction: A diagnostic accuracy study and proposed diagnostic pathway
Source: PLoS One. 2018 Nov 8;13(11):e0206946. doi: 10.1371/journal.pone.0206946 (PMC6224095; doi:10.1371/journal.pone.0206946)
Supplement: S1 Table — (DOCX) [file pone.0206946.s003.docx]

ETD-related symptoms:

- Feeling of fullness or pressure
- Otalgia
- Clicking on swallow / Toynbee
- Popping sensation
- Plugged / blocked / clogged sensation
- Hearing loss / muffled hearing
- Feeling of ‘cotton wool’ in ears
- Tinnitus / Ringing in ears
- Feeling of being ‘under-water’
- Squeaking or squelching
- Poor pressure equalisation / pain during flight, tunnel or dive
- Failed Valsalva (with explanation of the manouvre)
- Permanently feel like you have a cold
- Ear symptoms with a cold / sinusitis
- Ear symptoms better when lying down
- Ear symptoms worse with exercise or prolonged conversation
- Hearing own voice / a voice echo / autophony / like you are talking into a tunnel or barrel
- Hearing own breathing / loud breath sounds

S1 Table.
