# Supplementary material for: Eustachian tube dysfunction: A diagnostic accuracy study and proposed diagnostic pathway
Source: PLoS One. 2018 Nov 8;13(11):e0206946. doi: 10.1371/journal.pone.0206946 (PMC6224095; doi:10.1371/journal.pone.0206946)
Supplement: S2 Table — Variables are the same as those recorded in Table 4. Bivarate Pearson correlation (r) shown, p value (two tailed) indicated by shading: dark grey shading p<0.01, and light grey shading p<0.05. (DOCX) [file pone.0206946.s004.docx]

|  | Obs Val. | Rep Val. | TTAG Val. | Imp Val. | Obs Toyn. | Rep Toyn. | TTAG Toyn. | Imp. Toyn. | Sono-tub. | Tubo-man. | Tubo-imped. | Nine step | Tymp. | CETDA | ETDQ-7 | Panel  OETD |
| --- | --- | --- | --- | --- | --- | --- | --- | --- | --- | --- | --- | --- | --- | --- | --- | --- |
| Observed Valsalva | 1 | .627^**^ | .661^**^ | .551^**^ | .620^**^ | .366^**^ | .419^**^ | .435^**^ | .317^**^ | .335^**^ | .376^**^ | .254^**^ | -.237^*^ | -.026 | -.107 | .084 |
| Reported Valsalva | .627^**^ | 1 | .516^**^ | .424^**^ | .330^**^ | .475^**^ | .308^**^ | .341^**^ | .337^**^ | .187^*^ | .282^**^ | .177 | -.211^*^ | .017 | -.095 | .209^*^ |
| TTAG Valsalva | .661^**^ | .516^**^ | 1 | .697^**^ | .328^**^ | .140 | .314^**^ | .258^**^ | .131 | .283^**^ | .257^**^ | .060 | -.201^*^ | -.125 | -.192^*^ | -.045 |
| Impedance Valsalva | .551^**^ | .424^**^ | .697^**^ | 1 | .282^**^ | .101 | .339^**^ | .431^**^ | .189^*^ | .316^**^ | .420^**^ | .207^*^ | -.229^*^ | -.043 | -.082 | -.035 |
| Observed Toynbee | .620^**^ | .330^**^ | .328^**^ | .282^**^ | 1 | .639^**^ | .443^**^ | .522^**^ | .357^**^ | .366^**^ | .307^**^ | .354^**^ | -.256^**^ | -.014 | -.067 | .037 |
| Reported Toynbee | .366^**^ | .475^**^ | .140 | .101 | .639^**^ | 1 | .407^**^ | .433^**^ | .387^**^ | .238^*^ | .193^*^ | .415^**^ | -.185^*^ | -.011 | -.059 | .062 |
| TTAG Toynbee | .419^**^ | .308^**^ | .314^**^ | .339^**^ | .443^**^ | .407^**^ | 1 | .600^**^ | .386^**^ | .264^**^ | .289^**^ | .376^**^ | -.156 | -.145 | -.185^*^ | -.014 |
| Impedance Toynbee | .435^**^ | .341^**^ | .258^**^ | .431^**^ | .522^**^ | .433^**^ | .600^**^ | 1 | .486^**^ | .534^**^ | .512^**^ | .438^**^ | -.239^**^ | -.026 | -.079 | .083 |
| Sonotubometry | .317^**^ | .337^**^ | .131 | .189^*^ | .357^**^ | .387^**^ | .386^**^ | .486^**^ | 1 | .332^**^ | .359^**^ | .431^**^ | -.322^**^ | -.028 | -.029 | .134 |
| Tubomanometry | .335^**^ | .187^*^ | .283^**^ | .316^**^ | .366^**^ | .238^*^ | .264^**^ | .534^**^ | .332^**^ | 1 | .509^**^ | .304^**^ | -.316^**^ | .06 | .041 | .043 |
| Tuboimpedance | .376^**^ | .282^**^ | .257^**^ | .420^**^ | .307^**^ | .193^*^ | .289^**^ | .512^**^ | .359^**^ | .509^**^ | 1 | .272^**^ | -.380^**^ | .006 | .034 | .156 |
| Nine step | .254^**^ | .177 | .060 | .207^*^ | .354^**^ | .415^**^ | .376^**^ | .438^**^ | .431^**^ | .304^**^ | .272^**^ | 1 | -.322^**^ | .015 | .034 | .252^**^ |
| Tympanometry | .237^*^ | .211^*^ | .201^*^ | .229^*^ | .256^**^ | .185^*^ | .156 | .239^**^ | .322^**^ | .316^**^ | .380^**^ | .322^**^ | 1 | .122 | .128 | .168 |
| CETDA | -.026 | .017 | -.125 | -.043 | -.014 | -.011 | -.145 | -.026 | -.028 | .06 | .006 | .015 | -.122 | 1 | .872^**^ | .310^**^ |
| ETDQ-7 | -.107 | -.095 | -.192^*^ | -.082 | -.067 | -.059 | -.185^*^ | -.079 | -.029 | .041 | .034 | .034 | -.128 | .872^**^ | 1 | .253^**^ |
| Panel OETD | .084 | .209^*^ | -.045 | -.035 | .037 | .062 | -.014 | .083 | .134 | .043 | .156 | .152^*^ | -.168 | .310^**^ | .253^**^ | 1 |

S2 Table.
