# Supplementary material for: Eustachian tube dysfunction: A diagnostic accuracy study and proposed diagnostic pathway
Source: PLoS One. 2018 Nov 8;13(11):e0206946. doi: 10.1371/journal.pone.0206946 (PMC6224095; doi:10.1371/journal.pone.0206946)
Supplement: S3 Table — Variables are the same as those recorded in Table 4. Bivarate Pearson correlation (r) shown, p value (two tailed) indicated by shading: dark grey shading p<0.01, and light grey shading p<0.05. (DOCX) [file pone.0206946.s005.docx]

|  | Obs.  breath. | TTAG  breath. | Imp.  breath. | Sono.  breath. | Tuboman.  30 | Tuboman.  40 | Tuboman.  50 | CETDA | ETDQ-7 | Panel  PETD |
| --- | --- | --- | --- | --- | --- | --- | --- | --- | --- | --- |
| Observed breathing | 1 | .780** | .659** | .298** | .334** | .373** | .525** | .024 | .040 | .479** |
| TTAG breathing | .780** | 1 | .788** | .225* | .397** | .445** | .397** | .043 | .057 | .570** |
| Impedance breathing | .659** | .788** | 1 | .245** | .431** | .481** | .268** | .061 | .078 | .619** |
| Sonotubometry | .298** | .225* | .245** | 1 | .268** | .298** | -.031 | .167 | .162 | .169 |
| Tubomanometry 30 | .334** | .397** | .431** | .268** | 1 | .717** | .121 | .006 | .014 | .426** |
| Tubomanometry 40 | .373** | .445** | .481** | .298** | .717** | 1 | .334** | .019 | .005 | .479** |
| Tubomanometry 50 | .525** | .397** | .268** | -.031 | .121 | .334** | 1 | .037 | .066 | .299** |
| CETDA | .024 | .043 | .061 | .167 | .006 | .019 | .037 | 1 | .872** | .202* |
| ETDQ-7 | .040 | .057 | .078 | .162 | .014 | .005 | .066 | .872** | 1 | .188* |
| Panel PETD | .479** | .570** | .619** | .169 | .426** | .479** | .299** | .202* | .188* | 1 |

S3 Table.
