# Supplementary material for: Eustachian tube dysfunction: A diagnostic accuracy study and proposed diagnostic pathway
Source: PLoS One. 2018 Nov 8;13(11):e0206946. doi: 10.1371/journal.pone.0206946 (PMC6224095; doi:10.1371/journal.pone.0206946)
Supplement: S4 Table — If present, fatigue may be expected to affect either patient manoeuvre ability of test results. Continuous variables only assessed. Tubomanometry and Tuboimpedance were not assessed as differences at 30/40/50mbar are expected. (DOCX) [file pone.0206946.s006.docx]

| **Value** | **Manoeuvre** | **Unit** | **ANOVA p value** |
| --- | --- | --- | --- |
| **Test result value** | | | |
| TTAG | Valsalva | EAC press. (daPa) | 0.82 |
|  | Toynbee | ±range | 0.60 |
| Impedance | Valsalva | Eq vol (ml) | 0.40 |
|  | Toynbee |  | 0.86 |
| Sonotubometry | Swallow | Sound (dB) | 0.98 |
| **Nasopharyngeal pressure generated** | | | |
| TTAG | Valsalva | daPa | 0.96 |
|  | Toynbee | ±range daPa | 0.79 |
| Impedance | Valsalva | daPa | 0.88 |
|  | Toynbee | ±range daPa | 0.66 |

S4 Table.
