# Supplementary material for: Eustachian tube dysfunction: A diagnostic accuracy study and proposed diagnostic pathway
Source: PLoS One. 2018 Nov 8;13(11):e0206946. doi: 10.1371/journal.pone.0206946 (PMC6224095; doi:10.1371/journal.pone.0206946)
Supplement: S1 File — (DOCX) [file pone.0206946.s007.docx]

***Supporting Information***

**Calibration and Internal Validation Methods**

Calibration is the agreement between observed endpoints and predictions(19). Calibration was graphically assessed by plotting observed outcomes against predicted probabilities. Smooth nonparametric calibration curves were created by using a LOESS algorithm(20) to estimate the observed probabilities of OETD relative to the predicted probabilities. The calibration slope was estimated by logistic regression of the binary outcome (OETD present or not) on the linear predictor. The linear predictor, *L*, is the linear combination of the coefficients from the fitted model with the covariate values in the original sample, i.e. 𝐿 = 𝑎 + 𝑏_1_𝑥_1_ + 𝑏_2_𝑥_2_ + … + 𝑏_𝑖_𝑥_𝑖_, where 𝑎 is the model intercept, 𝑏_1_ to 𝑏_𝑖_ the set of regression coefficients, and 𝑥_1_ to 𝑥_𝑖_ the values of the *i* index tests.

Internal validation using bootstrapping was performed for both Clinical and Research models, and involved resampling with replacement from the original data set, thus replicating new samples similar to the original cohort; the size of each of the 200 samples was the same as the original data set. Each model was fitted to each bootstrap sample and the coefficients were then applied to the original sample to obtain a predicted probability of the occurrence of OETD for each patient. The *c*-statistic for the model was estimated and averaged over the 200 samples to obtain the optimism adjusted *c*-statistic.

**A description of Principle Component Analysis and Latent Class Analysis**

Principal Component Analysis (PCA) is a statistical approach used in exploratory data analysis to reduce a large set of possibly correlated variables (different tests and PROMs in our study) to a small set of variables that are linearly uncorrelated. These are called principle components. The first principal component accounts for as much of the variability in the data as possible, and each succeeding component accounts for as much of the remaining variability as possible. The relationship between each variable (test or PROM) and the components is expressed in the form of a correlation coefficient as a loading. In this way we explored which tests of ET function displayed common findings.

Latent class analysis (LCA) is used to assess diagnostic accuracy in situations where a reference standard diagnosis is imperfect or unavailable. This statistical technique combines multiple pieces of diagnostic information, in this case the test and PROM results, to determine disease status. Although disease status remains an unmeasured (“latent”) categorical variable, the study population is divided into two classes, disease positive and disease negative.
